# Atrial fibrillation ablation in patients with type 2 diabetes mellitus: long-term follow-up study for atrial tachyarrhythmia recurrence and adverse cardiovascular events

**DOI:** 10.1007/s11845-026-04380-5

**Published:** 2026-04-11

**Authors:** Mustafa Lütfullah Ardıç, Abdullah Eren Cetin, Hasan Burak Ozdemir, Fadime Koca, Hilmi Erdem Sumbul, Mevlut Koc

**Affiliations:** 1 Department of Cardiology, University of Health Sciences - Adana Health Practice and Research Center, Dr. Mithat Özsan Bulvarı Kışla Mah. 4522 Sok. No: 1 Yüreğir, Adana, Turkey; 2 Department of Cardiology, 25 Aralık State Hospital, Gaziantep, Turkey; 3 Department of Cardiology, Cukurova State Hospital, Adana, Turkey; 4 Department of Internal Medicine, University of Health Sciences - Adana Health Practice and Research Center, Adana, Turkey

**Keywords:** Atrial fibrillation, Type 2 diabetes mellitus, Cryoballoon ablation, Cardiovascular event

## Abstract

**Background:**

Type 2 diabetes mellitus (T2DM) is a modifiable risk factor both for atrial fibrillation (AF) and for the development of cerebrovascular-events in patients with AF. The aim of our study was to determine the effect of cryoballoon ablation (CBA) treatment on long-term atrial-tachyarrhythmia recurrence and adverse cardiovascular (CV) events in paroxysmal AF patients with T2DM.

**Methods:**

This retrospective-observational-cohort study included 748-patients (406-male,342-female,59.4 ± 11.8years) who underwent CBA with a diagnosis of paroxysmal AF between 2013−2023. The primary endpoint was atrial tachyarrhythmia recurrence (After-CBA≥3months) and the secondary endpoint was development of CV-events (CV mortality, non-fatal myocardial infarction and cerebrovascular-events). All patients were followed up for at least 24 months for atrial-tachyarrhythmia recurrence and CV event development.

**Results:**

The prevalence of T2DM was 26% in patients who underwent CBA. Atrial-tachyarrhythmia recurrence and CV events were more frequent in the T2DM group (24%vs15%,*p* = 0.004 and 33%vs9%,*p* = 0.045, respectively). Systolic-diastolic blood pressure, female gender, frequency of T2DM and anti-diabetic drug use, and left atrial diameter (LAd) values were higher in patients with atrial-tachyarrhythmia recurrence (*p* < 0.05). The presence of LAd and T2DM were shown to independently predict the development of atrial-tachyarrhythmia recurrence (OR = 1.235,95%CI-1.169-1.305, *p* < 0.001andOR = 1.843,95%CI-1.116-3.406,*p* = 0.030). Male-gender and presence of T2DM, CHA_2_DS_2_-VA score and uric-acid were shown to independently predict CV events (OR = 1.906,95%CI1.102-3.299,*p* = 0.021,OR = 2.148,95%CI-1.073-4.303,*p* = 0.013,OR = 1.613,95%CI-1.346-1.932,*p* = 0.001andOR = 1.322,95%CI-1.125-1.554,*p* = 0.001).

**Conclusion:**

The presence of T2DM is an important, independent and modifiable risk factor for both atrial-tachyarrhythmia recurrence and adverse CV events in patients treated with CBA for AF. It was concluded that comprehensive management of T2DM is necessary to improve long-term outcomes of CBA.

**Graphical Abstract:**

**Figure:**
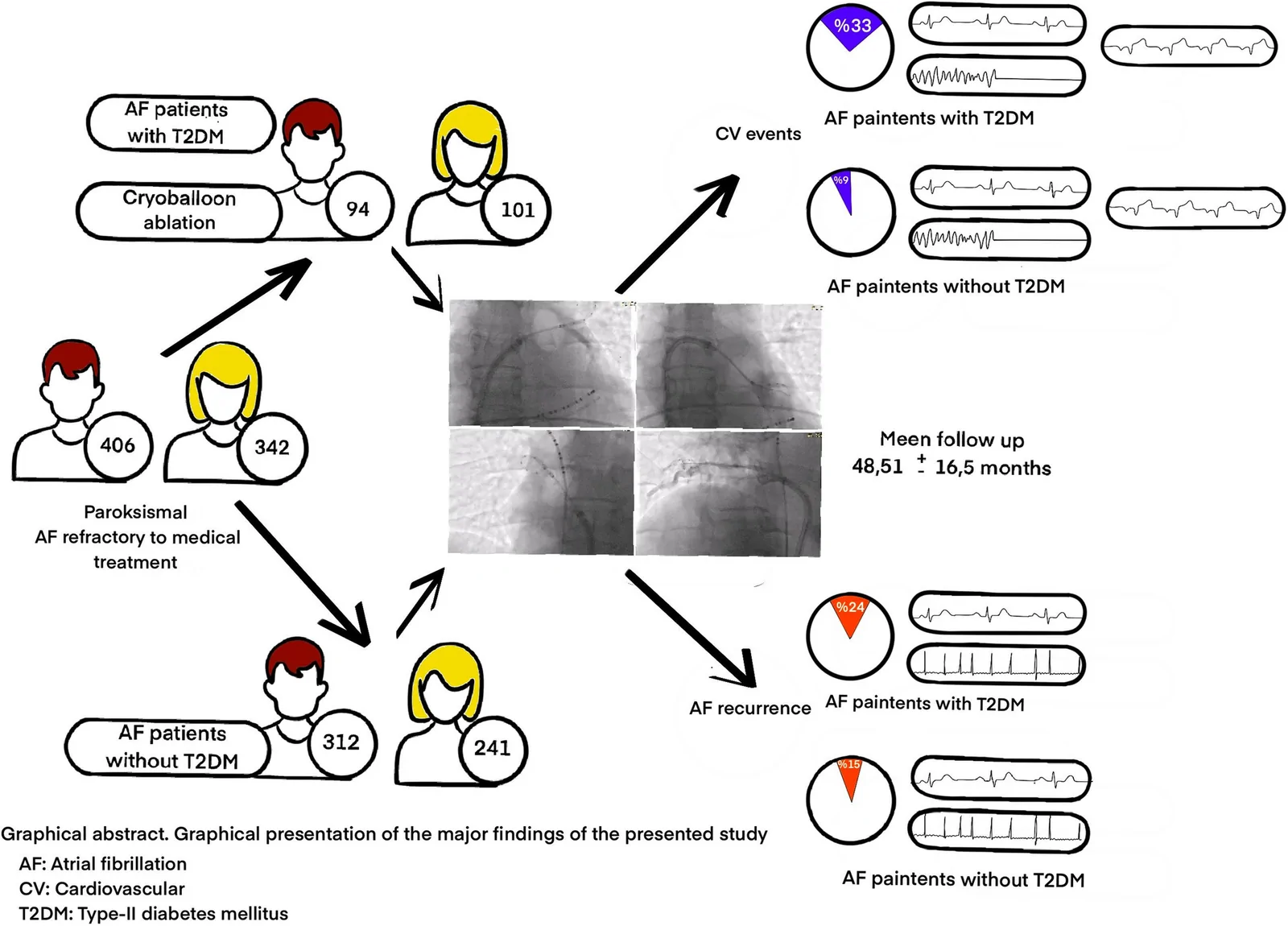

## Introduction

The increasing prevalence and incidence of both diabetes mellitus (DM) and atrial fibrillation (AF) worldwide increases the coexistence of these two important diseases. DM, which is an important risk factor for the development of AF, is also a very important global health problem, with a prevalence of 10.5% in the age range of 20 to 79 years, and up to 96% is Type 2 DM (T2DM) [1–3]. There is a bidirectional relationship between DM and AF; it has been reported that 25% of AF patients have DM, whereas 34% of patients with DM develop AF [1–4]. Also, the prognosis is even worse when these two diseases are associated [5–7]. In addition, the presence of DM in AF patients is a major risk factor for the development of thromboembolic events and is part of the CHA_2_DS_2_-VA score used in AF patients [1, 2].

As in the general population, catheter ablation is an effective and well-established treatment for symptomatic AF with DM and is recommended by ablation guidelines [1, 2]. There are several risk factors, mostly modifiable, associated with recurrence atrial tachyarrhythmia after catheter ablation therapy [2]. These risk factors can be summarized as DM, hypertension (HT), obesity, metabolic syndrome, physical inactivity, obstructive sleep apnea, alcohol consumption and smoking [1].

However, since the number of patients with T2DM included in AF ablation studies is relatively small and most of the studies are single-center, there are conflicting results regarding the net efficacy of ablation therapy, especially cryoballoon ablation (CBA), and its relationship with long-term recurrence and adverse cardiovascular (CV) events [4, 8–11]. Several studies have been conducted on the frequency of recurrence and related parameters after AF ablation in patients with DM, and while the presence of DM and recurrence were associated in the majority of these studies [4, 8, 10], it was reported that the presence of DM was not associated with AF recurrence in a registry report of 4429 patients [11]. Regardless of catheter ablation, the presence of DM in AF is associated with worsening quality of life, hospitalization and mortality [1, 2, 7]. The long-term prognostic significance of catheter ablation therapy in patients with DM is still unclear.

The aim of our study was to determine the effect of CBA treatment on long-term atrial tachyarrhythmia recurrence and adverse CV events in paroxysmal AF patients with T2DM.

## Methods

### Study population

**Fig. 1:**
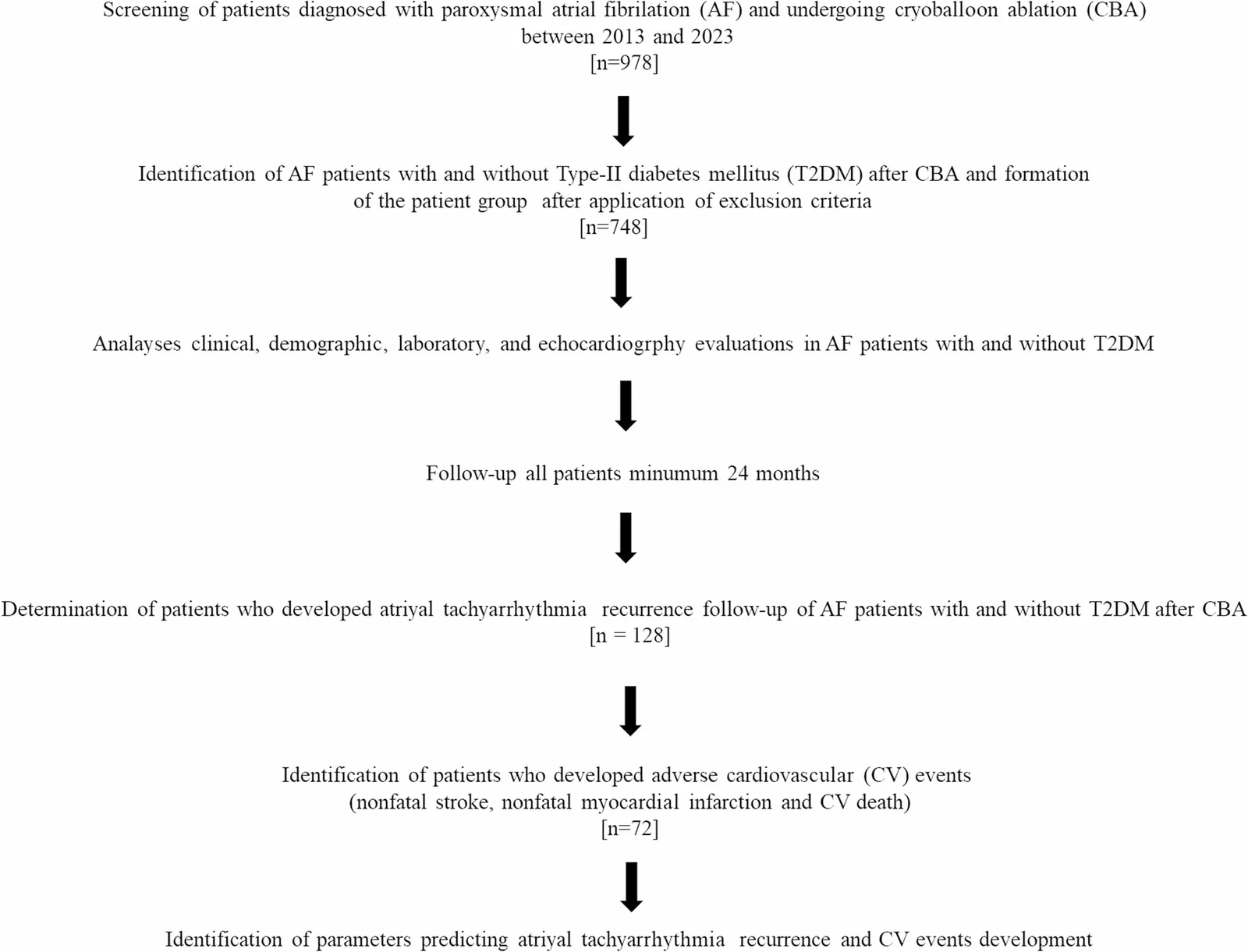
Study algorithm: (**a**) Identification of AF patients with and without T2DM after CBA and formation of the patient group after application of exclusion criteria [*n* = 748], (**b**) Analysis clinical, demographic, laboratory, and echocardiography evaluations in AF patients with and without T2DM, (**c**) Follow-up all patients minimum 24 months, (**d**) Determination of patients who developed AF recurrence follow-up of AF patients with and without T2DM after CBA [*n* = 128], (**e**) Identification of patients who developed CV events (nonfatal stroke, nonfatal myocardial infarction and CV death) [*n* = 72], (**f**) Identification of parameters predicting atrial tachyarrhythmia recurrence and CV events development

In this retrospective cohort study, patients who underwent CBA with a diagnosis of paroxysmal AF at the “University of Health Sciences - Adana Health Practice and Research Center - Department of Arrhythmia Electrophysiology Laboratory” between 2013 and 2023 were screened. It was determined that a total of 978 patients with PAF underwent CBA. To determine the number of patients to be included in the study, power analysis was performed considering previous studies and their results (80% power and *p* < 0.05). After this analysis, it was found that approximately 240 patients were sufficient to be included in the study. Those who had CBA failure, had complications during CBA, had insufficient information for CV events and atrial tachyarrhythmia recurrence during follow-up, ≤ 18 years of age, persistent and permanent AF, pregnant and < 3 months post-pregnancy, chronic inflammatory disease, chronic kidney disease (eGFR < 60 mL/min/1.73m^2^), obstructive sleep apnea syndrome, severe aortic or mitral valve disease;, known systolic and diastolic heart failure (HF), hematologic disease, active thyroid disease, chronic liver disease and active cancer were excluded from the study. The study algorithm is shown in Fig. 1 and graphical abstract.

On the day the patients were hospitalized for CBA, their anamnesis and physical examination were noted. All risk factors used in the calculation of CHA_2_DS_2_-VA score were evaluated individually. Age, congestive HF, HT, DM, history of previous cerebrovascular event and presence of vascular disease were recorded. Systolic - diastolic blood pressures and heart rate were obtained. The CHA_2_DS_2_-VA score was calculated with the previously mentioned parameters as specified in the European Heart Association 2024 AF guidelines [1]. (1) C−Congestive HF [1 point], (2) H− HT [1 point], (3) A−Age ≥ 75 [2 points], (4) D− DM [1 point], S−History of stroke [2 points], (6) V−History of vascular disease [1 point], (7) A−Age 65–74 [1 point]. Anti-arrhythmic, anti-coagulant and anti-diabetic treatments of the patients were recorded.

Laboratory parameters of all patients on the day of CBA were recorded. Complete blood count, fasting plasma glucose, serum blood urea nitrogen (BUN), creatinine, estimated glomerular filtration rate (eGFR), sodium, potassium, uric acid, total cholesterol, low-density lipoprotein cholesterol, high-density lipoprotein cholesterol and triglycerides were measured. Glycated hemoglobin (HbA1c) level was measured only in patients with T2DM. All patients underwent M-mode and two-dimensional transthoracic echocardiographic evaluation in the echocardiography laboratory prior to CBA. Examinations were performed using an EPIQ 7 C ultrasound system (Philips Healthcare, 3000 Minuteman Road, Andover, MA, USA). All measurements were obtained in accordance with the recommendations of the European Association of Cardiovascular Imaging and the American Society of Echocardiography [12].

The M-mode cursor was positioned just above the tips of the mitral valve leaflets, and measurements were acquired with the left ventricular (LV) axis oriented perpendicular to the ultrasound beam. LV volumes and left ventricular ejection fraction (LVEF) were calculated using the biplane Simpson’s method from apical two-chamber and apical four-chamber views. Left atrial diameter (LAd) was measured from parasternal long-axis views, and end-systolic LA dimensions were recorded.

### Atrial fibrillation diagnosis and follow-up

For the diagnosis of AF, 12-lead electrocardiography, 72-hour Holter electrocardiography examination or the Telemetry system available in the Arrhythmia service were used according to the latest ESC AF guideline criteria [1, 2]. Accordingly, (i) irregular atrial activity and atrial cycle length variability (shorter than 200 ms) (ii) irregular R-R interval (iii) absence of recurrent prominent P waves (iv) rapid, irregular fibrillation waves of different shape and size instead of P waves, (v) finally, irregular and variable ventricular rate was considered as AF. For the diagnosis of paroxysmal AF, it was evaluated by two electrophysiologists (MLA and AEC) blinded to patient information. As a result of this evaluation, in case of discordant results between two electrophysiologists (interobserver), the final decision was made by the third electrophysiologist in our clinic (MK).

### Atrial fibrillation cryoballoon ablation protocol

All patients underwent AF ablation after at least 8 h of fasting while continuing an effective oral anti-coagulant therapy. The procedure was performed under sedoanalgesia using a combination of midazolam and fentanyl. Using the Seldinger technique, 8 F, 6 F and 6 F sheaths were placed in the right femoral vein, right femoral artery and vein, respectively. A coronary sinus diagnostic catheter was placed through the left femoral vein. The right femoral artery was used during pressure monitoring and non-coronary cusp pigtail catheter placement during septostomy. Blood required for ACT measurements was also obtained from this sheath. The 8 F right femoral vein sheath was then replaced with a preface 8.5 F long sheath. A single septostomy was then performed under fluoroscopy and pressure guidance using the HeartSpan Transseptal Needle (MeritMedicalTM) septostomy needle. After septostomy, a 28-mm cryoballoon catheter (Arctic Front Advance Pro™; the fourth-generation cryoballoon, Medtronic, Inc., Minneapolis, MN, ABD ve POLARx™ ST; Boston Scientific, Marlborough, Ma, USA) was combined with an intraluminal mapping catheter (Achieve™; Medtronic, Inc. ve POLARMAP™, Boston Scientific and gently advanced into the left atrial (LA) cavity through a 15-Fr steerable sheath (FlexCath™; Medtronic, Inc. and POLARSHEATH™, Boston Scientific). Cryoablation of the upper left, lower left, lower right, lower right and upper right pulmonary veins was planned first, followed by upper left, lower left, lower right and upper right pulmonary veins, respectively. First, pulmonary veins were selected with an intraluminal mapping catheter, then a cryoballoon catheter was advanced over the mapping catheter and inflated. Optimal occlusion of the inflated cryoballoon in each PV antrum was confirmed using opaque material injection in the scope, and a mapping catheter was placed in the optimal position to obtain PV potential recordings for real-time monitoring of PV isolation. Then cryoenergy was given. Cryoenergy was continued for 180 s if the PV’s time to isolation was shorter than 30 s and for 240 s if the PV’s time to isolation was ≥ 30 s. Bonus freeze was not applied. During right-sided CBA, to prevent phrenic nerve palsy, diaphragm stimulation was performed using a diagnostic catheter placed in the coronary sinus for right renic nerve stimulation. Cryoablation success was considered as disappearance of pulmonary vein signals and demonstration of pulmonary vein entry and PV exit block. Adenosine/ATP testing was not routinely performed. After all procedures, it was checked whether PV isolation continued at 20 min. For veins without PV isolation, cryoenergy was applied again.

### Follow-up: atrial tachyarrhythmia recurrence and adverse cardiovascular events

The primary endpoint of the study was determined as the development of atrial tachyarrhythmia recurrence. The secondary endpoint was determined as the development of adverse CV events. The first 3 months following CBA were considered the blanking period, during which all antiarrhythmic medications (amiodarone, flecainide, and propafenone) were continued. After the blanking period, antiarrhythmic drug therapy was discontinued in all patients. All patients were followed up for atrial tachyarrhythmia recurrence at 3-month intervals for at least 24 months after inclusion in the study. The presence of atrial tachyarrhythmia (AF or atrial flutter) of > 30 s documented by 12-lead electrocardiography or Holter electrocardiography during follow-up (except for the first 3 months in AF ablation patients) was considered atrial tachyarrhythmia recurrence. In case of atrial tachyarrhythmia recurrence, 2nd ablation procedure was performed. Anti-arrhythmic therapy was given to patients who did not want repeat ablation treatment due to atrial tachyarrhythmia recurrence. Patients with persistent AF at follow-up were enrolled. All patients were followed up similarly for at least 24 months for adverse CV events. CV mortality, non-fatal myocardial infarction and non-fatal cerebrovascular events were defined as adverse CV events.

### Statistical analysis

All analyses were performed using SPSS 23.0 (SPSS for Windows 20.0, Chicago, IL, USA). Continuous variables in the group data were expressed as mean ± standard deviation and categorical variables as number and percentage. The “Shapiro-Wilk” test was used to assess whether the distribution of continuous variables was normal. In the comparison of countable parameters between the 2 groups, Student T test and Mann Whitney U test were used according to normal and non-normal distribution, respectively. Chi-square test was used to compare categorical data. All parameters that were significant in univariate analysis (*p* < 0.05) were evaluated by multivariate logistic regression analysis to identify patients with atrial tachyarrhythmia recurrence and adverse CV events. Statistical significance was defined as a p value < 0.05 for all comparisons.

## Results

Of the 978 patients who underwent successful CBA with a diagnosis of PAF in our clinic, 748 (77%) patients were included after exclusion criteria (406 males, 342 females, age 59.4 ± 11.8 years). It was determined that 195 (26%) of the patients included in the study had T2DM. All patients were followed up for at least 24 months (mean 48.51 ± 16.5 months) for atrial tachyarrhythmia recurrence and CV event development. During the follow-up period, the incidence of atrial tachyarrhythmia recurrence and CV events was 128 (17.1%) and 72 patients (9.6%), respectively.

Among the patients with atrial tachyarrhythmia recurrence, 48 were managed with medical therapy, whereas the remaining 80 patients underwent electrophysiological study and three-dimensional RF ablation due to recurrent atrial tachyarrhythmia.

In cases with CV events, the number of −nonfatal stroke nonfatal myocardial infarction− CV death was 20 − 27 − 25, respectively. Cohen kappa values that evaluate the interobserver variability were over 90% for all measurements (*p* < 0.001). Patients included in the study were grouped as with−without atrial tachyarrhythmia recurrence and with−without CV events and all parameters were compared.

### Clinical, demographic, treatment and follow-up findings of the patients with and without T2DM

Clinical, demographic, treatment and follow-up data of patients with and without T2DM are shown in Table 1. The frequency of HF, HT, vascular disease and cerebrovascular event, use of beta-blockers, calcium channel blockers, ACE inhibitors or ARBs, anti-coagulants, anti-diabetics and insulin, atrial tachyarrhythmia recurrence and CV events were significantly higher in patients with T2DM compared to patients without T2DM. In addition, age, systolic and diastolic blood pressures and CHA_2_DS_2_-VA score were higher in T2DM patients. Other clinical, demographic, treatment and follow-up data were similar between the two groups (Table 1).

**Table 1:** Clinical, demographic, treatment and follow-up findings of the patients with and without T2DM

Variables	AF patients with T2DM *n* = 195	AF patients without T2DM *n* = 553	*p*
Age (year)	65.7 ± 7.8	58.1 ± 12	**0.001**
Gender (Male), n	94 (48%)	312 (56%)	0.054
Heart failure, n (%)	31 (16%)	44 (8%)	**0.002**
Hypertension, n (%)	128 (66%)	273 (49%)	**0.001**
Vascular disease, n (%)	67 (34%)	106 (19%)	**< 0.001**
Previous cerebrovascular accident, n (%)	12 (6%)	1 (5%)	**< 0.001**
Systolic blood pressure (mmHg)	137 ± 13	134 ± 15	**0.009**
Diastolic blood pressure (mmHg)	84 ± 11	78 ± 11	**0.005**
Heart rate (beat/min)	85 ± 12	86 ± 17	0.879
CHA_2_DS_2_-VA score 0‒1‒2‒3‒4‒5‒6‒7‒8, n	3.03 ± 1.59 0‒30‒55‒46‒32‒18‒7‒3‒4	1.15 ± 1.12 187‒187‒117‒40‒18‒3‒0‒1‒0	**< 0.001**
Propafenone use, n (%)	43 (22%)	130 (24%)	0.767
Flecainide use, n (%)	42 (21%)	144 (26%)	0.248
Amiodarone use, n (%)	58 (30%)	166 (30%)	1.000
Beta blocker or CCB use, n (%)	185 (95%)	434 (78%)	**< 0.001**
ACE inhibitor or ARB use, n (%)	114 (59%)	241 (44%)	**< 0.001**
Oral anti-coagulant use, n (%)	161 (83%)	267 (48%)	**< 0.001**
Oral anti-diabetic use, n (%)	166 (85%)	0 (0%)	**< 0.001**
Insulin use, n (%)	35 (18%)	0 (0%)	**< 0.001**
Atrial tachyarrhythmia recurrence, n (%)	46 (24%)	82 (15%)	**0.004**
Cardiovascular events, n (%)	24 (33%)	48 (9%)	**0.045**

The values were shown as mean ± standard deviation or n (%), *T2DM* Type 2diabetes mellitus, *AF* Atrial fibrillation, *CCB* Calcium channel blockers, *ACE* Angiotensin converting enzyme, *ARB* Angiotensin receptor blockers, statistically significant p values were shown in bold

### Laboratory findings of the patients with and without T2DM

Laboratory data of the patient groups with and without T2DM are shown in Table 2. Fasting plasma glucose, creatinine, BUN, uric acid levels and LAd diameter were significantly higher in patients with T2DM compared to patients without T2DM. In addition, hemoglobin and eGFR levels of T2DM patients were lower than those of non-T2DM patients. Other laboratory data were similar between the two groups (Table 2).

**Table 2:** Laboratory findings of the patients with and without T2DM

Variables	AF patients with T2DM *n* = 195	AF patients without T2DM *n* = 553	*p*
White blood cell (10³/µL)	7.51 ± 2.4	7.32 ± 2.2	0.894
Platelet count (10³/µL)	252 ± 70	245 ± 79	0.272
Hemoglobin (g/dL)	13.0 ± 1.7	13.5 ± 1.4	**< 0.001**
Fasting plasma glucose (mg/dL)	147 ± 59	94 ± 17	**< 0.001**
HbA1c (%)	6.65 ± 1.4	−	**−**
Creatinine (mg/dL)	0.93 ± 0.48	0.76 ± 0.28	**< 0.001**
Blood urea nitrogen (mg/dL)	38.9 ± 19	28.1 ± 9.2	**< 0.001**
eGFR (mL/min/1.73 m^2^)	79.3 ± 18	97.6 ± 16	**< 0.001**
Uric acid (mg/dL)	5.43 ± 1.5	4.85 ± 1.47	**< 0.001**
Sodium (mmol/L)	138 ± 2.1	139 ± 2.2	0.661
Potassium (mmol/L)	4.35 ± 0.43	4.27 ± 0.40	0.291
Total cholesterol (mg/dL)	190 ± 44	193 ± 48	0.533
HDL cholesterol (mg/dL)	48 ± 15	50 ± 14	0.191
LDL cholesterol (mg/dL)	126 ± 34	128 ± 34	0.512
Triglycerides (mg/dL)	171 ± 86	165 ± 92	0.388
LVEF (%)	56.8 ± 6.7	58.9 ± 4.5	**< 0.001**
LA dimension (mm)	40.8 ± 4.2	37.2 ± 4.3	**< 0.001**

The values were shown as mean ± standard deviation or n (%), *T2DM* Type 2diabetes mellitus, *AF* Atrial fibrillation, *eGFR* Estimated glomerular filtration rate, *HDL* High-density lipoprotein, *LDL* Low-density lipoprotein, *LVEF* Left ventricular ejection fraction, *LA* Left atrium, statistically significant p values were shown in bold

### Clinical, demographic, treatment and laboratory findings associated with atrial tachyarrhythmia recurrence

Patients included in the study were divided into two groups as atrial tachyarrhythmia recurrence and non-recurrence after follow-up. It was found that systolic blood pressure, diastolic blood pressure and LAd diameter were significantly higher in patients who developed atrial tachyarrhythmia recurrence compared to those who did not develop atrial tachyarrhythmia recurrence (Table 3). In addition, the presence of T2DM, oral anti-diabetic use and female gender were significantly higher in those who developed atrial tachyarrhythmia recurrence (Table 3). HbA1c levels were found to be similar in patients with and without atrial tachyarrhythmia recurrence (6.67 ± 1.3 vs. 6.64 ± 1.5 and *p* = 0.919). Furthermore, the proportion of patients with HbA1c level > 7% was similar in patients with and without atrial tachyarrhythmia recurrence (33% vs. 35% and *p* = 0.461). Other clinical, demographic, treatment, follow-up and laboratory data were similar in the groups with and without atrial tachyarrhythmia recurrence (*p* > 0.05).

**Table 3:** Clinical, demographic, treatment and laboratory findings associated with atrial tachyarrhythmia recurrence

Variables	Atrial tachyarrhythmia recurrence (+) *n* = 128	Atrial tachyarrhythmia recurrence (-) *n* = 620	*p*
Systolic blood pressure (mmHg)	138 ± 16	134 ± 15	**0.008**
Diastolic blood pressure (mmHg)	82 ± 13	79 ± 12	**0.015**
Type 2 diabetes mellitus, n (%)	46 (36%)	149 (24%)	**0.004**
Gender (Female), n	68 (53%)	274 (44%)	**0.040**
Oral anti-diabetic use, n (%)	39 (31%)	127 (21%)	**0.011**
Left atrium dimension (mm)	41.6 ± 4.6	37.5 ± 4.3	**< 0.001**

The values were shown as mean ± standard deviation or n (%),statistically significant p values were shown in bold

### Clinical, demographic, treatment and laboratory findings associated with adverse CV events

After follow-up, the patients included in the study were divided into two groups as with and without adverse CV events. Age, male gender, T2DM, HF, previous cerebrovascular event, HT and vascular disease frequency, CHA_2_DS_2_-VA score, fasting plasma glucose, creatinine, BUN and uric acid levels were found to be significantly higher and eGFR levels were significantly lower in the group with CV events compared to those without CV events (Table 4). Other clinical, demographic, treatment, follow-up and laboratory data were similar in the groups with and without CV events (*p* > 0.05).

**Table 4:** Clinical, demographic, treatment and laboratory findings associated with adverse CV events

Variables	Adverse CV events (+) *n* = 72	Adverse CV events (-) *n* = 676	*p*
Age (year)	65.1 ± 10	58.8 ± 12	**< 0.001**
Gender (Male), n (%)	49 (68%)	357 (53%)	**0.009**
Type 2 diabetes mellitus, n (%)	24 (33%)	169 (24%)	**0.045**
Heart failure, n (%)	16 (22%)	59 (9%)	**< 0.001**
Previous cerebrovascular accident, n (%)	7 (10)	8 (1)	**< 0.001**
Hypertension, n (%)	52 (72%)	349 (52%)	**0.001**
Vascular disease, n (%)	30 (42%)	143 (21%)	**< 0.001**
CHA_2_DS_2_-VA score 0‒1‒2‒3‒4‒5‒6‒7‒8, n	2.72 ± 1.93 8‒11‒20‒13‒6‒7‒3‒3‒1	1.52 ± 1.41 179‒206‒152‒73‒44‒14‒4‒1‒3	**< 0.001**
Fasting plasma glucose (mg/dL)	125 ± 57	102 ± 35	**0.048**
Creatinine (mg/dL)	0.95 ± 0.45	0.79 ± 0.39	**0.006**
Blood urea nitrogen (mg/dL)	35.9 ± 18	30.1 ± 13	**0.015**
eGFR (mL/min/1.73 m^2^)	86.4 ± 25	93.5 ± 19	**0.019**
Uric acid (mg/dL)	5.93 ± 2.1	4.90 ± 1.4	**< 0.001**

The values were shown as mean ± standard deviation or n (%), *CV* Cardiovascular, *eGFR* Estimated glomerular filtration rate, statistically significant p values were shown in bold

### Multivariate logistic regression analysis to identify patients with atrial tachyarrhythmia recurrence

**Fig. 2:**
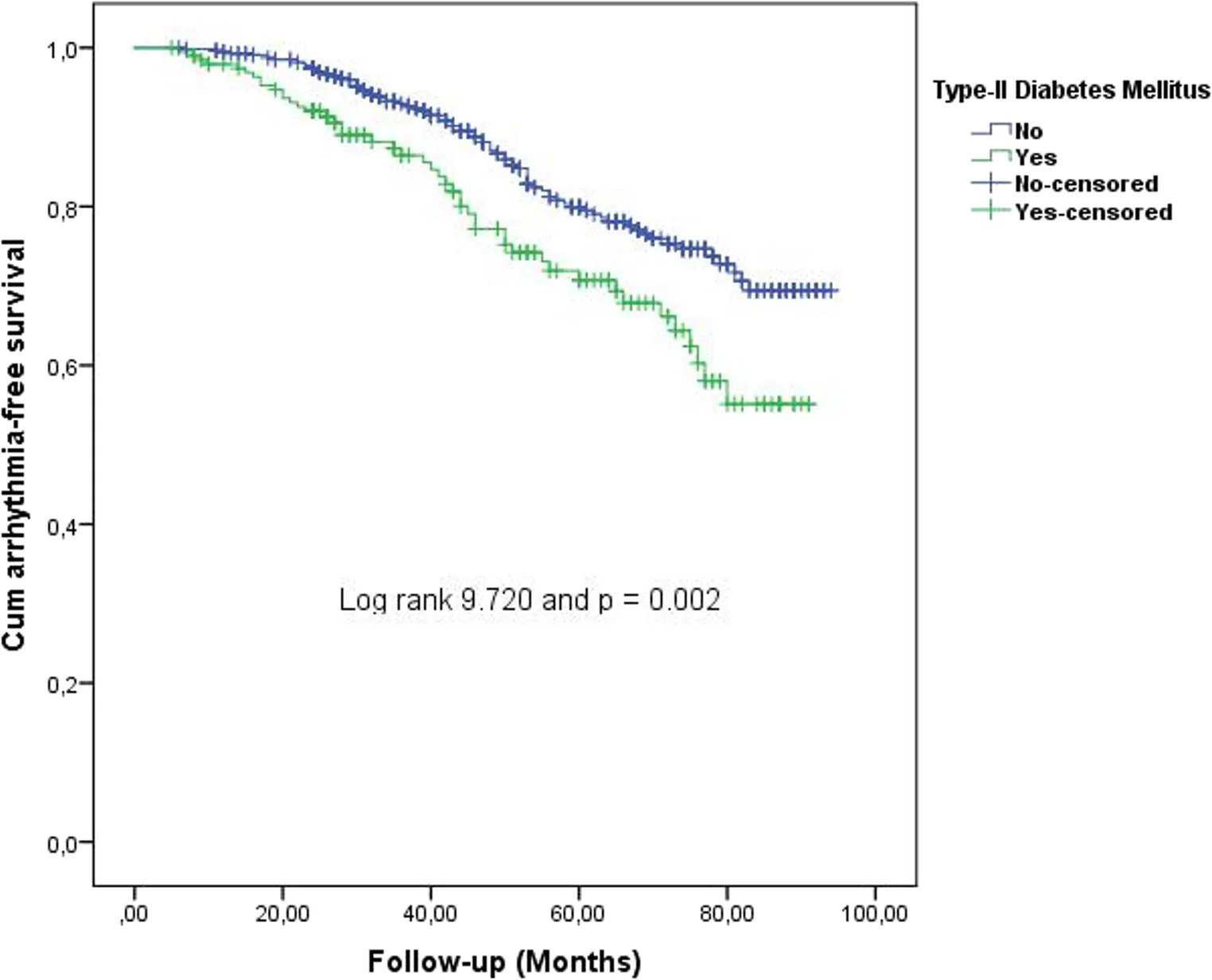
Kaplan–Meier survival curves for AF patients with arrhythmias-free survival: presence or absence of Type 2 diabetes mellitus. The curves were constructed with the Kaplan–Meier method and the p-value was computed via log-rank test

Multivariate logistic regression analysis was performed to identify the parameters associated with atrial tachyarrhythmia recurrence in univariate analysis as independent predictors of patients with atrial tachyarrhythmia recurrence (Table 5). As a result of this analysis, it was determined that the parameters that best determined the development of atrial tachyarrhythmia recurrence were the increase in LAd diameter and the presence of T2DM, respectively (Table 5). The Kaplan–Meier survival curves in Fig. 2 show that the presence of T2DM was significantly related to atrial tachyarrhythmia recurrence in patients with AF.

**Table 5:** Multivariate logistic regression analysis to identify patients with atrial tachyarrhythmia recurrence

	Odds Ratio	95% CI	*p*
Gender (Female), *n*	1.218	0.816 − 1.820	0.334
Type 2 diabetes mellitus, (presence)	1.843	1.116 − 3.406	**0.030**
Systolic blood pressure (mmHg)	1.014	0.999 − 1.030	0.074
Diastolic blood pressure (mmHg)	1.003	0.984 − 1.024	0.734
Oral anti-diabetic use (presence)	1.109	0.427 − 2.876	0.832
Left atrium dimension (each 1 mm)	1.235	1.169 − 1.305	**< 0.001**

Statistically significant p values were shown in bold

### Multivariate logistic regression analysis to identify patients with adverse CV events

**Fig. 3:**
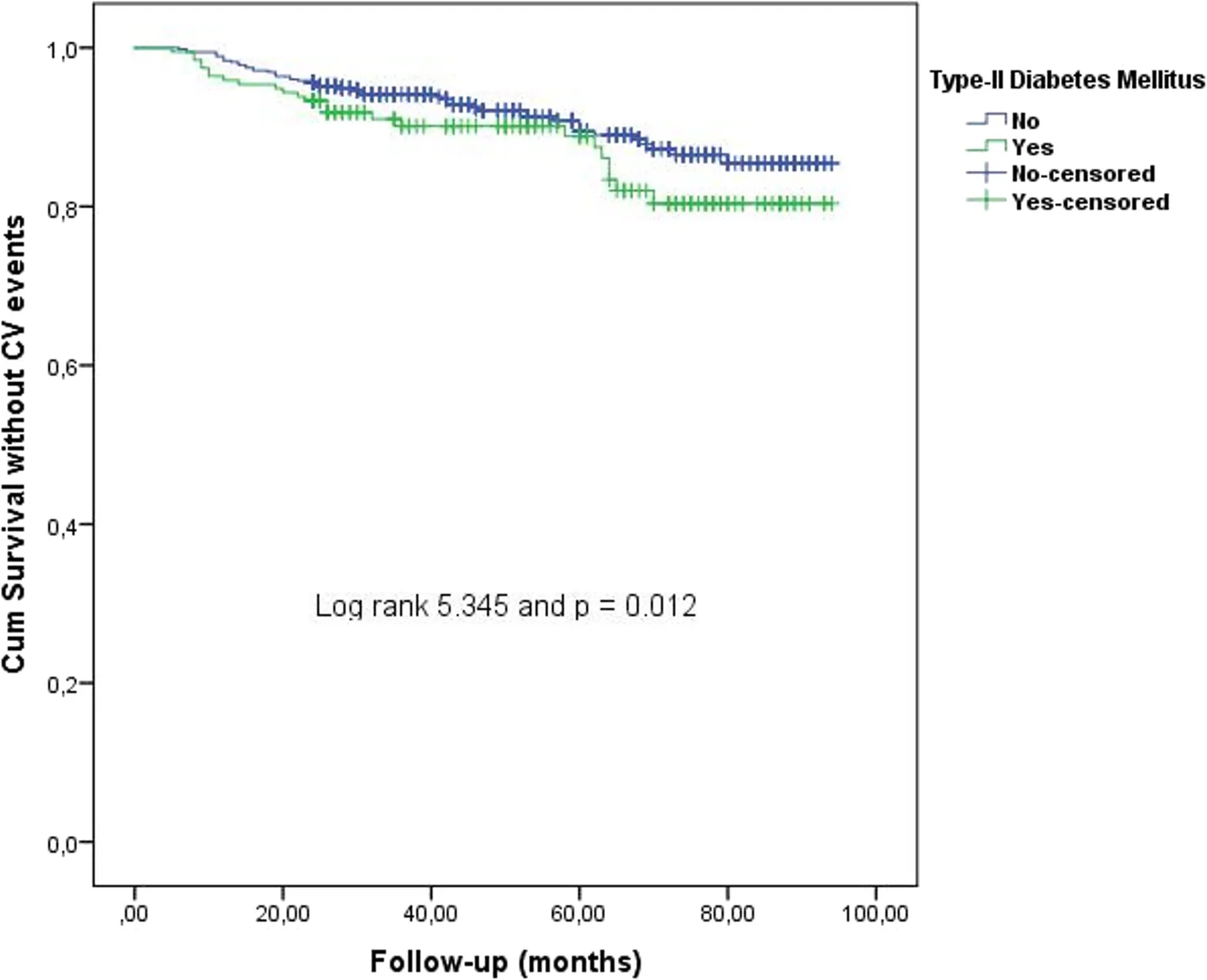
Kaplan–Meier survival curves for AF patients with long-term adverse cardiovascular events: presence or absence of Type 2 diabetes mellitus. The curves were constructed with the Kaplan–Meier method and the p-value was computed via log-rank test

Multivariate logistic regression analysis was performed to determine the independent predictors of CV events among the parameters associated with CV events in the univariate analysis (Table 6). As a result of this analysis, the presence of T2DM and male gender, CHA_2_DS_2_-VA score and uric acid level were found to be the best predictors of CV event development (Table 6). According to this analysis, male gender and presence of T2DM were higher odds of CV events by 1.91 and 2.15 times, respectively. Similarly, in the same analysis, each 1 CHA_2_DS_2_-VA score and 1 mg/dL increase in uric acid were higher odds of CV events by 61% and 32%, respectively. The Kaplan–Meier survival curves in Fig. 3 show that the presence of T2DM was significantly related to adverse CV events in patients with AF.

**Table 6:** Multivariate logistic regression analysis to identify patients with adverse cardiovascular events

	Odds Ratio	95% CI	*p*
Age (each year)	1.029	0.987 − 1.073	0.179
Gender (Male), n (%)	1.906	1.102 − 3.299	**0.021**
Type 2 diabetes mellitus (presence)	2.148	1.073 − 4.303	**0.013**
Heart failure (presence)	1.410	0.503 − 3.947	0.514
Previous cerebrovascular accident, n (%)	0.330	0.056 − 1.943	0.220
Hypertension (presence)	0.780	0.338 − 1.800	0.560
Vascular disease (presence)	0.786	0.339 − 1.826	0.576
CHA_2_DS_2_-VA score (each 1)	1.613	1.346 − 1.932	**0.001**
Plasma glucose (each mg/dL)	1.004	0.998 − 1.010	0.145
Creatinine (each 1 mg/dL)	1.302	0.696 − 2.435	0.408
Blood urea nitrogen (each 1 mg/dL)	1.009	0.997 − 1.023	0.272
eGFR (each mL/min/1.73 m^2^)	1.012	0.994 − 1.031	0.197
Uric acid (each 1 mg/dL)	1.322	1.125 − 1.554	**0.001**

## Discussion

The main findings of this study can be summarized as follows: (1) up to 26% of patients with PAF undergoing CBA have T2DM and this finding is consistent with the literature (25%); (2) atrial tachyarrhythmia recurrence (24% vs. 15%) and adverse CV events (33% vs. 9%) after CBA are more frequent in patients with T2DM; (3) the presence of T2DM is independently associated with long-term atrial tachyarrhythmia recurrence and adverse CV events after CBA; (4) the presence of T2DM after CBA increases atrial tachyarrhythmia recurrence by 84.3% and adverse CV event development by 2.15-fold.

Many studies have confirmed that the presence of DM is an independent risk factor for the development of AF [1–4]. The pathophysiological relationship between AF and DM is complex and multifactorial and is associated with autonomic dysfunction, atrial electrical, electromechanical and structural remodeling [13]. It has been shown that increased advanced glycation end products and reactive oxygen species in patients with DM result in fibrotic changes, ion channel and gap junction remodeling in the atria [13, 14]. As a result of these changes, conduction heterogeneity increases, conduction velocity decreases, action potential duration is prolonged and the electrophysiologic environment leading to the development of AF is created [1, 2]. The prevalence of DM in patients with AF has been reported to be 25% [2]. In our study, in accordance with the literature, the prevalence of DM was found to be 26% in patients undergoing CBA treatment. However, to the best of our knowledge, the majority of previous studies comparing AF ablation results in patients with and without DM included a lower proportion of patients with DM (18.5% [4], 7.7% [11], 9.3% [8] and 12.8% [10]). Therefore, it was thought that our study might be more compatible with real life data.

Unfortunately, significant rates of atrial tachyarrhythmia recurrence occur in CBA studies in patients with paroxysmal AF, as previously demonstrated by us [1, 2, 15, 16]. There are many risk factors associated with recurrence after AF ablation, the most important of which can be summarized as AF type and duration, age and presence of other CV risk factors, medical treatment, experience of the ablation team and duration of follow-up [1, 2]. In most of the studies investigating atrial tachyarrhythmia recurrence after ablation in the DM subgroup, atrial tachyarrhythmia recurrence was reported to be higher in patients with DM compared to those without DM [4, 8, 10]. However, in a large registry report of 4429 patients, atrial tachyarrhythmia recurrence was reported to be similar in patients with and without DM [11]. Similarly, in a non-randomized observational study in 7 European centers, it was reported that the presence of DM was associated with recurrence in patients with persistent AF, whereas similar atrial tachyarrhythmia recurrence was reported in patients with paroxysmal AF with and without DM [8]. Long-term atrial tachyarrhythmia recurrence frequency in DM patients has been reported to be between 19 and 36% [4, 8, 10, 11, 17]. In our study, atrial tachyarrhythmia recurrence after ablation was shown to be higher in patients with T2DM with a rate of 24% and our finding was consistent with the majority of previous studies. Our most important difference from previous studies was that the CBA method was used in all cases in our study and all cases were paroxysmal AF. In our study, long-term atrial tachyarrhythmia recurrence was evaluated in 195 T2DM patients with paroxysmal AF, whereas the number of paroxysmal AF patients in previous studies similar to our study was 111 [8], 21 [4], 35 [10] and only 211 patients were evaluated in the German ablation registry report [11]. In our clinic, we use CBA in all patients with paroxysmal AF and radiofrequency ablation in persistent, long-standing persistent and recurrent atrial tachyarrhythmia.

Apart from the fact that the presence of DM after catheter ablation predicts atrial tachyarrhythmia recurrence [4, 8, 10], several studies have shown that the most important risk factor associated with atrial tachyarrhythmia recurrence in patients with DM is glycemic control [17, 18]. Therefore, the 2024 EHRA AF ablation guideline also recommends an HbA1c level of < 7% and adherence to dietary modification for long-term rhythm preservation after AF ablation in patients with DM [1, 2]. In the study conducted by Donnellan et al. [18], it was reported that the frequency of atrial tachyarrhythmia recurrence was 2-fold higher in patients with HbA1c > 9% compared to patients with HbA1c < 7%. In the same study, multivariate analysis showed that a > 10% decrease in HbA1c level before AF ablation was the most important predictor of arrhythmia-free survival [18]. Therefore, optimal glycemic control is very important in patients with DM who will undergo AF. In a study conducted a decade ago evaluating atrial tachyarrhythmia recurrence in 1464 patients with DM who underwent AF ablation, HbA1c level was shown to independently determine atrial tachyarrhythmia recurrence [17].

In a study similar to our study in the literature, Wang A et al. [4] included 351 patients with 65 T2DM with good glycemic control (median HbA1c 6.8%) and showed that HbA1c level was high in patients with atrial tachyarrhythmia recurrence but not closely associated with the development of atrial tachyarrhythmia recurrence [4]. In the same study, 56.9% recurrence occurred after 29.5 months of follow-up, similar to our study, and age, presence of HT and DM (2.24-fold) and LAd independently determined the recurrence [4]. In our study, similar to the previous study, the median HbA1c level was 6.3% in patients with DM and it was shown that it was similar in patients with and without atrial tachyarrhythmia recurrence. Furthermore, similar to the previous study, we showed that the presence of T2DM and LAd diameter values independently determined the development of atrial tachyarrhythmia recurrence. In conclusion, our study demonstrated that the presence of DM increased atrial tachyarrhythmia recurrence despite good glycemic control before CBA in DM patients with AF.

Apart from the frequency of coexistence of DM and AF, the development of adverse CV events over time is higher when both diseases are present in the same patient [1–6]. To the best of our knowledge, there is insufficient data in the literature on the development of adverse CV events after AF ablation in patients with and without long-term arrhythmia-free survival. In the German ablation registry report, it was reported that similar rates of adverse CV events occurred in patients with and without DM after ablation [11]. This is why our study is valuable. We found that the development of adverse CV events was higher in patients with DM compared to those without DM during a follow-up period of 48.51 ± 16.5 months and DM increased the development of adverse CV events by 2.15 times. We also showed in our study that the development of atrial tachyarrhythmia recurrence after AF ablation independently determines the development of CV events.

### Limitations

This study has several important limitations. The study was single-center and retrospective. Prospective, randomized, multicenter studies with more patients may be more significant. In our study, only paroxysmal AF cases were included and persistent, longstanding persistent and permanent AF cases were excluded. Patients who underwent CBA treatments were included in our study. New method PWA and RF ablation procedures in AF ablation treatment were not included. Therefore, persistent AF and PWA - RF ablation protocols may also need to be tried to make our findings more valuable. In particular, it has been clearly demonstrated that exercise, weight loss and dietary recommendations can prevent atrial tachyarrhythmia recurrence [1, 2]. In our study, we did not evaluate the effect of diet, physical activity and exercise on atrial tachyarrhythmia recurrence and CV events. Similarly, metformin, sodium-glucose co-transporter 2 inhibitor and glucagon-like peptide 1 receptor agonists used in T2DM have been shown to reduce atrial tachyarrhythmia recurrence [19–21]. The use of these drugs and their effect on follow-up parameters were not evaluated in our study. Previous studies have reported similar intraoperative complications during catheter ablation in patients with and without DM [4, 8, 11]. We included successful and complication-free CBA cases in our study. In our study, HbA1c level was measured only in patients with T2DM; this measurement was not performed in patients with normal glucose metabolism due to social security institution limitations. If HbA1c was measured in the entire study, there might have been an association between atrial tachyarrhythmia recurrence and HbA1c. Atrial tachyarrhythmia recurrence was assessed using 48-hour Holter ECG monitoring. Longer monitoring durations, such as 3–7 days, might have provided more accurate detection of recurrence.

Moreover, we did not analyze several procedural parameters recorded during CBA that may influence atrial tachyarrhythmia recurrence, including time to isolation of the pulmonary veins (PVs), total cryoenergy delivery time, lowest temperature achieved, balloon warming angle, and time to reach the nadir temperature [22]. The inclusion of these parameters could have provided a more comprehensive understanding of the determinants of recurrence and strengthened the interpretability of our findings.

In addition, patients with diabetic nephropathy or chronic kidney disease (eGFR < 60 mL/min/1.73 m²)—conditions that are clinically important in the management and prognosis of patients with AF and diabetes mellitus—were defined as exclusion criteria in our study. Therefore, the findings of our study may not be generalizable to all patients with AF.

## Conclusion

The presence of T2DM is an important, independent and modifiable risk factor for both atrial tachyarrhythmia recurrence and adverse CV events in patients treated with CBA for paroxysmal AF. It was concluded that comprehensive management of T2DM is necessary to improve long-term outcomes of CBA. However, it was concluded that the results obtained in our study should be supported by multicenter, randomized studies with a much larger number of patients in order for the results obtained in our study to be clinically usable and more advanced.

## Data Availability

For ethical and administrative reasons, the data could not be made available.

## Abbreviations

AF: Atrial fibrillation
BUN: Blood urea nitrogen
CBA: Cryoballoon ablation
CV: Cardiovascular
DM: Diabetes mellitus
ECG: Electrocardiography
eGFR: Glomerular filtration rate
EHRA: European Heart Rhythm Association
HbA1c: Glycated hemoglobin
HF: Heart failure
HT: Hypertension
LA: Left atrial
LAd: Left atrial diameter
LVEF: Left ventricular ejection fraction
TTI: Time to isolation
T2DM: Type 2diabetes mellitus
